# Analysis of CASP12 diagnostic and prognostic values in cervical cancer based on TCGA database

**DOI:** 10.1042/BSR20192706

**Published:** 2019-12-16

**Authors:** Guo Feng, Zhang Beilei, Chen Caizhi, Zou Wen

**Affiliations:** 1 Department of Oncology of the Second Xiangya Hospital of Central South University, Changsha, China; 2 Department of Gynecology of Hunan Provincial Cancer Hospital of Central South University, Changsha, China

**Keywords:** cervical cancer, diagnosis, prognosis, TCGA

## Abstract

The present study aims to find a differential protein-coding gene caspase 12 (*CASP12*) in cervical cancer (CC) based on the (TCGA) database and verify its clinical diagnostic and prognostic values. The transcriptome and clinicopathological data of CC were downloaded from the TCGA database and through screening, we found that PDE2A and CASP12 were independent prognostic factors for CC patients. According to the median expression, the patients were divided into groups with high and low CASP12 and PDE2A expression. There was no difference in survival between PDE2A high and low expression groups (*P*=0.099), whereas there was a significant difference between CASP12 high and low expression groups (*P*=0.033). The serum from 68 CC patients (experimental group) and 50 healthy people (control group) was collected to detect the relative expression of CASP12 using qRT-PCR and plotted the ROC curve. The relative expression of CASP12 in the experimental group was significantly lower than in the control group (*P*<0.05). The area under the curve (AUC) of CASP12 was 0.865. There were statistically significant differences between CASP12 groups with high and low expression in terms of differentiation, lymph node metastasis, tumor size, FIGO staging, and clinical outcomes (*P*<0.05), but not in terms of age, HPV types and pathological types (*P*>0.05). The 3-year survival in the CASP12 low expression group was significantly worse than in the CASP12 high expression group (*P*=0.028). In conclusion, the expression level of CASP12 can be used as a diagnostic and prognostic biomarker for patients with CC.

## Introduction

Worldwide, cervical cancer (CC) is the fourth deadliest cancer among women [1]. According to a study in 2018 [2], there were more than 570000 new CC patients in the world and over 300000 deaths in the same period. Another study showed that China had 98000 new CC patients and 30500 new deaths in 2015 [3]. This disease has become more and more common in young females [4,5]. Unapparent and easy to be ignored at an early stage, but when patients are admitted to a hospital, CC is already at a progressive stage. Therefore, patients usually miss the best treatment timeframe. Previous studies have shown [6,7] that surgical treatment in patients with early CC have a 5-year survival rate of 97.5% and do not need later adjuvant treatment, which reduces cost, side effects and improves survival time and quality of life. Therefore, early diagnosis of CC is essential. However, there are only a few diagnostic biomarkers with high specificity for early CC.

Major oncology research programs have been launched to improve next-generation sequencing technology [8,9]. Among them, the Cancer Genome Atlas (TCGA) is the most important. It is designed to draw the genome map of human tumors through large-scale high-throughput technologies for genome sequencing and DNA chip, find new treatments of cancers and improve diagnosis and prevention by exploring the development, progression and potential molecular mechanisms of tumors [10,11]. The present study aims to screen differentially expressed genes in CC based on the TCGA database and verify their clinical diagnostic and prognostic values by collecting and detecting the serum from patients with CC and healthy people, find potential diagnostic biomarkers, and provide references for clinicians.

## Materials and methods

### Data sources

The gene expression data of CC in the TCGA database and the patients’ clinical data were downloaded from the Broad Institute’s Genome Data Analysis Center (GDAC) Firehose (http://gdac.Broadinstitute.org/). The data were collected from the TCGA repository (http://can-cergenome.nih.gov/cancergenomics/tissuesamples), sequenced and analyzed by standardized treatment schemes. Altogether, 306 cancer and 3 matched paracancerous sample data were obtained using IlluminaHiSeq2000. Perl scripts were used to combine the files into mRNA-symbol matrix files, including protein-coding genes, long non-coding RNA and pseudogenes.

### Collection of clinical samples

Sixty-eight patients with CC treated in our hospital from January 2015 to February 2016 were enrolled in the experimental group. These patients had an average age of 51.2 ± 8.2 years, and all the clinicopathological characteristics were collected. Fifty healthy women who underwent a physical examination in our hospital were enrolled in the control group. Their average age was 50.1 ± 8.5 years.

#### Inclusion criteria

Patients who met FIGO staging [12]; patients with pathology diagnosis of CC; patients with complete clinical data and patients who signed an informed consent form.

#### Exclusion criteria

Patients who received radiotherapy and chemotherapy; patients with other tumors; patients with severe cardio-cerebral dysfunction; patients who did not cooperate with follow-up visits and patients with immune deficiencies.

There was no statistically significant difference in age between the two groups (*P*>0.05).

### Preprocessing of the TCGA database

Patients whose survival time was less than 90 days were excluded. The log_2_ conversion was performed on the gene expression because the gene expression of a single sample was <1 but >0. Also, the log_2_(X+1) conversion was performed on samples to make the data similar to the gene expression because the conversion results could be negative [13]. Relevant information was extracted from clinical files, including patient ID, survival time, survival condition, clinical staging and gene expression of CC patients. The gene expression files were combined with the matrix files and named differentially expressed gene files. Other data were deleted due to excessive loss.

### Detection of mRNA expression

Five milliliters of venous blood was collected from fasting patients in the morning. The blood was submerged for 30 min and centrifuged at 3000 rpm for 10 min to obtain a supernatant. TRIzol (Invitrogen; Thermo Fisher Scientific, Inc., U.S.A.) was used to extract the total RNA from the serum. Ultraviolet spectrophotometer and agarose gel electrophoresis were used to detect its purity, concentration and integrity. The total RNA was reverse transcribed into cDNA using 5× TransScript® All-in-One SuperMix for qPCR and gDNA Remover, with the steps carried out following the manufacturer’s kit. The cDNA was stored, part of which was taken for subsequent experiments. A 7900PCR instrument from ABI was used for PCR amplification based on TransScript Two-Step RT-PCR SuperMix (TransGen Biotech, Beijing, China, AQ201-01) kit. The system was as follows: 1 μl cDNA, each 0.5 μl upstream and downstream primers, 12.5 μl of 2× TransTaq® HIFI PCR SuperMix II and Nuclease-free water added up to 25 μl. The conditions were as follows: pre-denaturation at 94°C for 30 s, denaturation at 94°C for 5 s, and annealing at 60°C for 30 s. Each sample was provided with the same three wells, and the experiment was conducted thrice. GAPDH was used as an internal reference for caspase 12 (CASP12), and 2^−ΔΔ*C*_t_^ was used to analyze the data. CASP12 primers were designed and synthesized by Shanghai GenePharma Co., Ltd. The upstream and downstream primer sequences of CASP12 were 5′-TTCAACAACCGTAACTGCCAGAGTC-3′ and 5′-CTGTCAGTGGTGAACCAAACAATCC-3′. Those of GAPDH were 5′-CACCCACTCCTCCACCTTTG-3′ and 5′-CCACCACCCTGTTGTTGTAG-3′.

### Follow-up

Patients’ survival was followed up to 3 years by telephone at the 1st, 3rd, 6th, 9th, 12th, 15th, 18th, 21st, 24th, 30th and 36th months after discharge, respectively.

### Statistical analysis

The edgeR package in R was used to analyze the differential expression of sample genes in the TCGA database, and the data met the requirements of *P*<0.001 and fold change = 4. We performed univariate and multivariate Cox regression analyses on differentially expressed gene files. SPSS 20.00 edition was used to analyze the data statistically, Graph Pad, 7 to plot figures. K–S test was used to analyze data distribution, and data confirming normal distribution was tested using a *t* test, while comparison between groups was made using independent sample *t* test. The Pearson χ^2^ test was used to assess the association between CASP12 expression and clinicopathological parameters. Kaplan–Meier and Log-rank tests were used to analyze the survival rate, and the ROC curve to plot the diagnostic value of CASP12 in CC. CASP12 has a diagnostic value in CC when the area under the curve (AUC) is >0.5. *P*<0.05 indicates a statistical difference.

**Figure 1:**
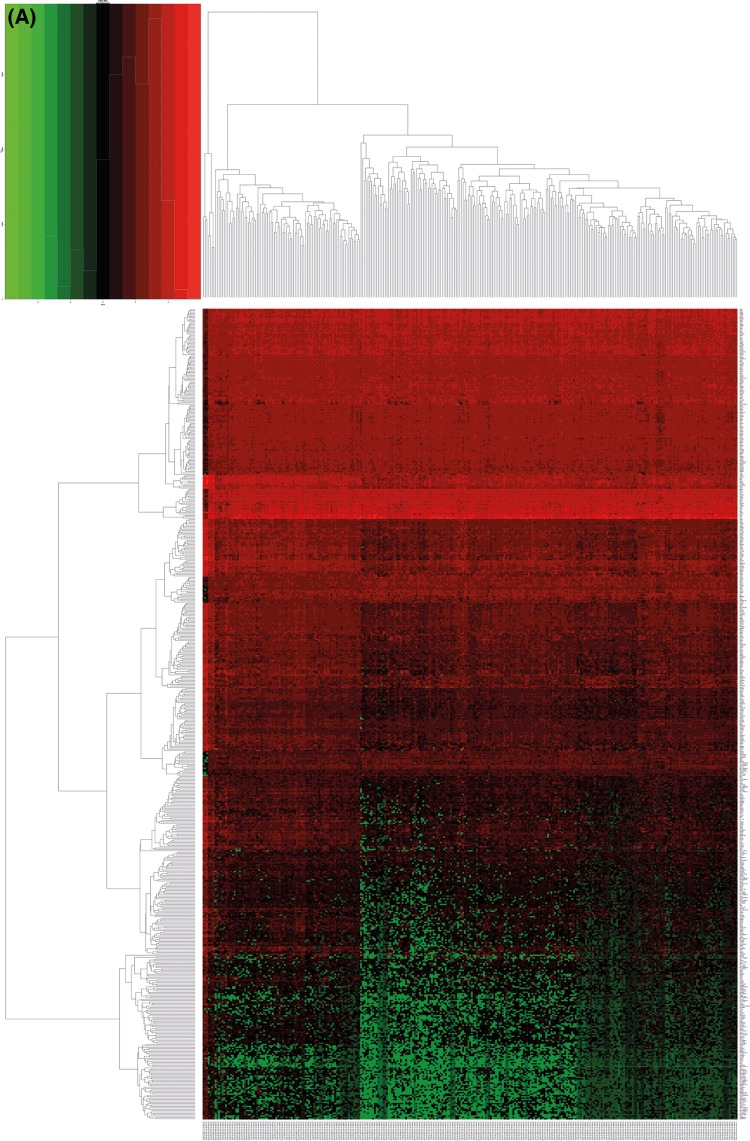

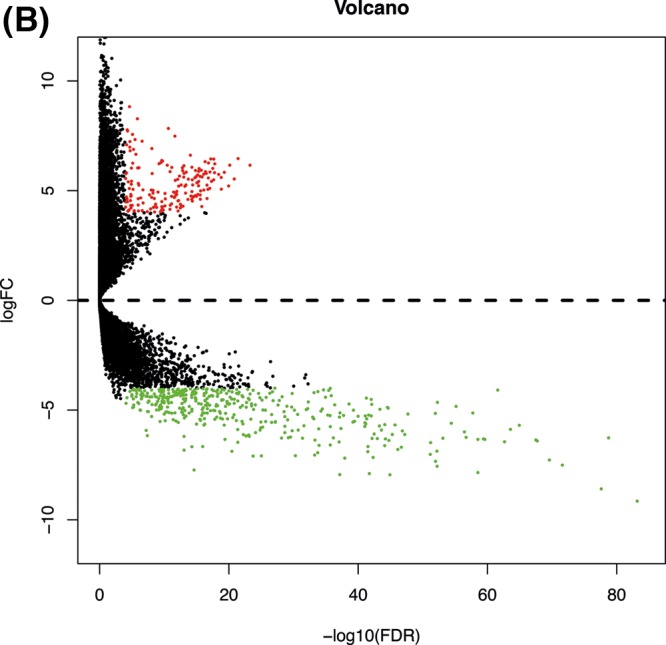
Differential expression genetic map of CC in the TCGA database (**A**) It is a heatmap. Red represents high expression, and green represents a low expression. (**B**) It is a volcano plot. The x-axis is the logarithm of −log_10_ after correction. The larger the value, the more significant the difference. The y-axis is log_2_ (FC), and the larger the absolute value, the greater the fold change.

## Results

### Differentially expressed genes in the TCGA database

In the present study, 590 differentially expressed genes were found using screening, 180 were highly expressed and 410 were lowly expressed. Five high and low expressed genes, with the most significant difference, are shown in Table 1 and Figure 1.

**Table 1:** Five high and low expressed genes with the most significant difference

Expression		Log FC	Log CPM	*P*-value	FDR
High gene					
	TROAP	6.163528308	5.442794884	2.74E-26	5.48E-24
	KIF18B	6.46370624	5.329511551	2.23E-24	3.78E-22
	CDC6	5.529731275	5.816794217	9.33E-24	1.49E-21
	NEK2	6.157637092	5.292400179	5.05E-23	7.42E-21
	CDCA8	5.215753068	5.610514593	7.61E-23	1.09E-20
Low gene					
	PGM5-AS1	−9.150190886	−1.360099552	2.11E-88	6.83E-84
	TRPC4	−6.266668467	0.062122961	1.13E-83	1.82E-79
	TCEAL6	−8.593210953	−2.459793695	2.29E-82	2.46E-78
	CNN1	−7.50634589	5.380915681	3.16E-76	2.55E-72
	PTGER3	−7.275089869	1.983684539	3.97E-74	2.56E-70

### Cox analysis of survival

**Figure 2:**
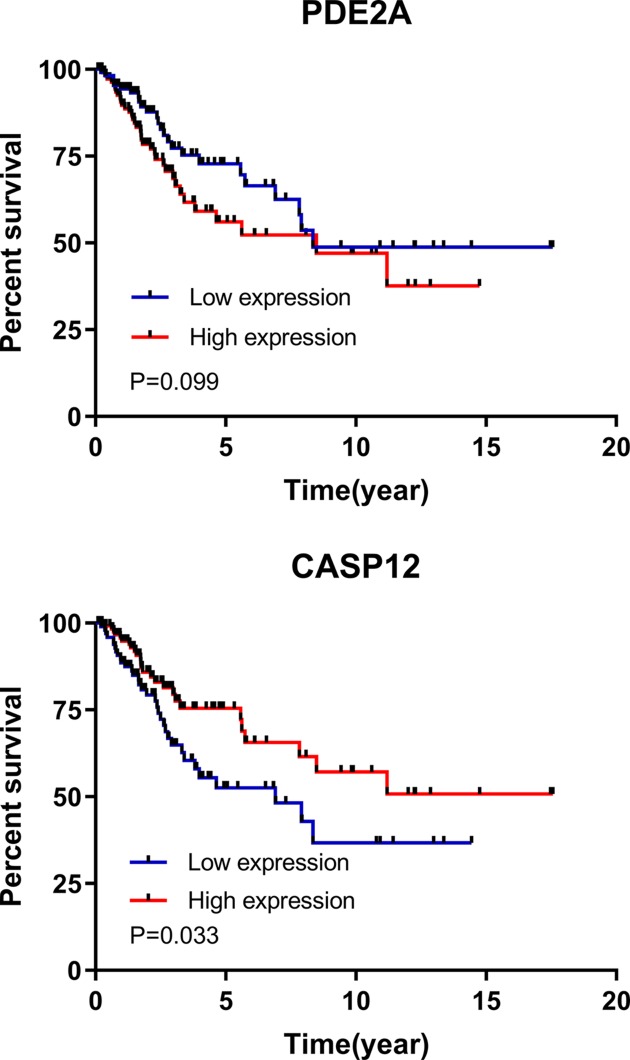
Survival in high and low expression groups There was no difference in survival between the PDE2A groups with high and low expression (*P*=0.099), whereas there was a significant difference between the CASP12 groups with high and low expression (*P*=0.033).

According to the univariate Cox regression analysis, there were 36 factors with differences. From those, ten with the most significant differences were CENPM, NTRK3, CD300LG, PTTG1, KIAA0101, PCP4, CASP12, CLEC3B, HAND2 and TP73. According to the multivariate analysis of the 36 factors, PDE2A and CASP12 with differences were independent prognostic factors for patients with CC. The patients were divided into high and low expression groups according to the median expression of PDE2A and CASP12, and the survival curves were plotted. There was no difference in survival between the PDE2A groups with high and low expression (*P*=0.099), whereas there was a significant difference between the CASP12 groups with high and low expression (*P*=0.033). More details are shown in Tables 2 and 3, and Figure 2.

**Table 2:** Univariate Cox regression analysis of the ten factors

Factors	β	SE	Z-value	*P*-value	HR	Lower .95	Upper .95
CENPM	−5.496	0.004	−4.746	0.000	0.004	0.000	0.040
NTRK3	−0.565	0.568	−3.332	0.001	0.568	0.407	0.792
CD300LG	−0.508	0.602	−3.305	0.001	0.602	0.445	0.813
PTTG1	−5.351	0.005	−3.237	0.001	0.005	0.000	0.121
KIAA0101	−2.843	0.058	−3.200	0.001	0.058	0.010	0.332
PCP4	−0.390	0.677	−3.104	0.002	0.677	0.530	0.866
CASP12	−0.515	0.598	−3.030	0.002	0.598	0.428	0.834
CLEC3B	−0.947	0.388	−3.019	0.003	0.388	0.210	0.717
HAND2	−0.551	0.576	−2.999	0.003	0.576	0.402	0.826
TP73	−1.124	0.325	−2.930	0.003	0.325	0.153	0.689

**Table 3:** Multivariate Cox regression analysis of the two factors

Factors	β	SE	Z-value	*P*-value	HR	Lower .95	Upper .95
PDE2A	2.720	0.855	3.179	0.001	15.177	2.838	81.167
CASP12	−0.853	0.294	−2.900	0.004	0.426	0.239	0.758

### Expression and diagnostic value of CASP12 mRNA

**Figure 3:**
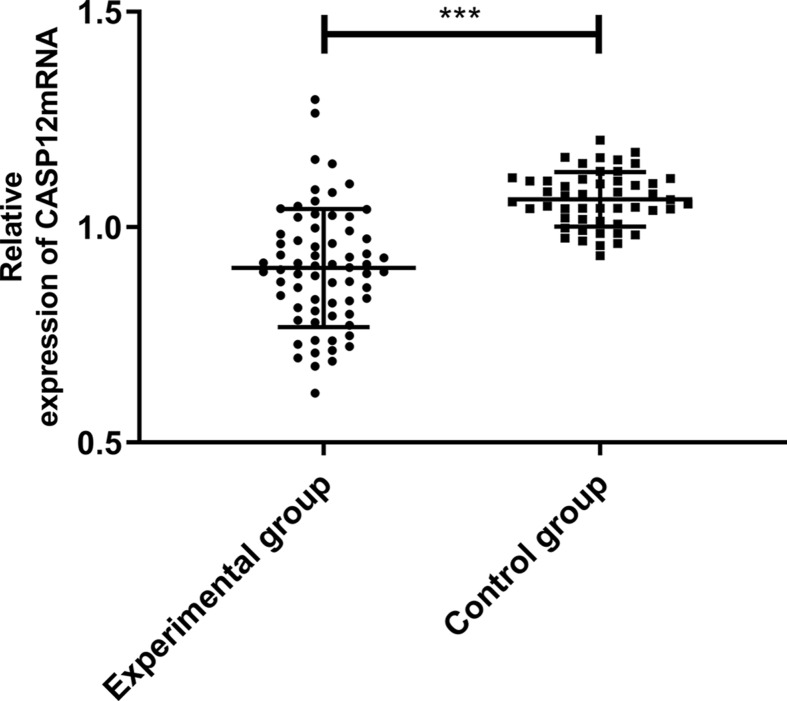
Comparison of the relative expression of CASP12 mRNA The relative expression of CASP12 mRNA in the experimental group (patients with CC) was significantly lower than in the control group (healthy people). ***Indicates *P*<0.001.

**Figure 4:**
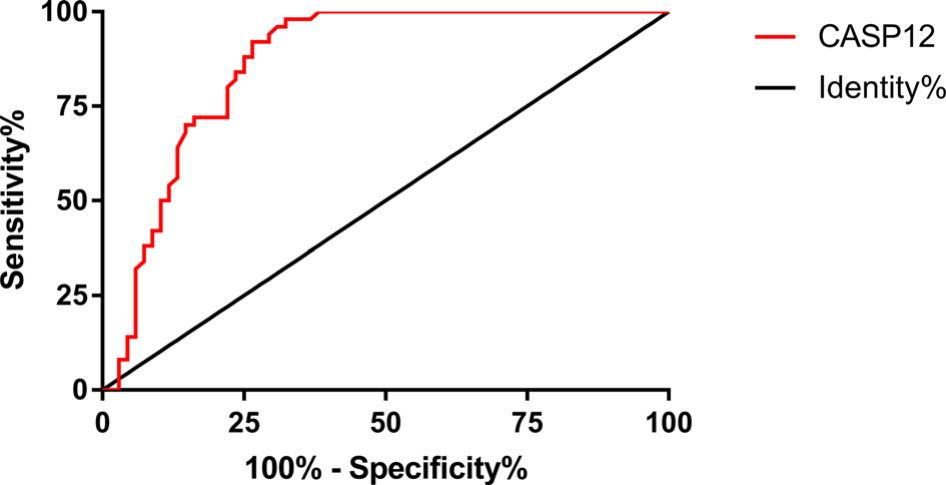
ROC curve of CASP12 in CC

According to the qRT-PCR, the relative expression of CASP12 in the experimental group (patients with CC) was significantly lower than in the control group (healthy people) (*P*<0.05). More details are shown in Figure 3. According to the ROC curve, the AUC of CASP12 was 0.865, 95CI%: 0.799–0.932, the specificity was 67.64%, and the sensitivity was 98.00%, with a Youden index of 65.65%, and a cut-off value > 0.959. More details are shown in Figure 4.

### Comparison of clinical data

According to the median expression of CASP12, the patients were divided into high (*n*=34) and low (*n*=34) expression groups. Then the correlation between the levels of CASP12 and clinicopathological parameters was analyzed to determine the clinical relevance of CASP12 expression in CC. The results showed that CASP12 expression was significantly associated with differentiation, lymph node metastasis, tumor size, FIGO staging and clinical outcomes (*P*<0.05), but not with age, HPV types and pathological types (*P*>0.05). More details are shown in Table 4.

**Table 4:** Relationship between CASP12 mRNA expression level by groups and clinical data of patients with CC [*n*(%)]

Factor		High expression group (*n*=34)	Low expression group (*n*=34)	χ^2^//Z value	*P*-value
Age					
	>50 years old	13 (38.24)	8 (23.53)	1.722	0.189
	≤50 years old	21 (61.76)	26 (76.47)		
FIGO staging					
	I–IIa	22 (64.71)	4 (11.76)		
	IIb–IV	12 (32.95)	30 (88.24)	20.176	0.001
Differentiation					
	Poor	4 (11.76)	12 (35.29)	9.571	0.008
	Medium	13 (38.24)	16 (47.06)		
	High	17 (50.00)	6 (17.65)		
Lymph node metastasis				5.231	0.022
	Yes	4 (11.76)	12 (35.29)		
	No	30 (88.24)	22 (64.71)		
HPV types					
	Type 16	21 (61.76)	23 (67.65)		
	Type 18	6 (17.65)	6 (17.65)	0.535	0.911
	Others	5 (14.71)	4 (11.76)		
	Negative	2 (5.88)	1 (2.94)		
Pathological types				1.705	0.426
	Squamous carcinoma	28 (82.36)	24 (70.58)		
	Adenocarcinoma	4 (11.76)	5 (14.71)		
	Adeno-squamous carcinoma	2 (5.88)	5 (14.71)		
Tumor size				5.314	0.021
	>4 cm	2 (5.88)	9 (26.47)		
	≤4 cm	32 (94.12)	25 (73.53)		
Clinical outcomes					
	Survival	29 (85.29)	21 (61.76)	4.836	0.028
	Death	5 (14.71)	13 (38.24)		

### Patient survival

**Figure 5:**
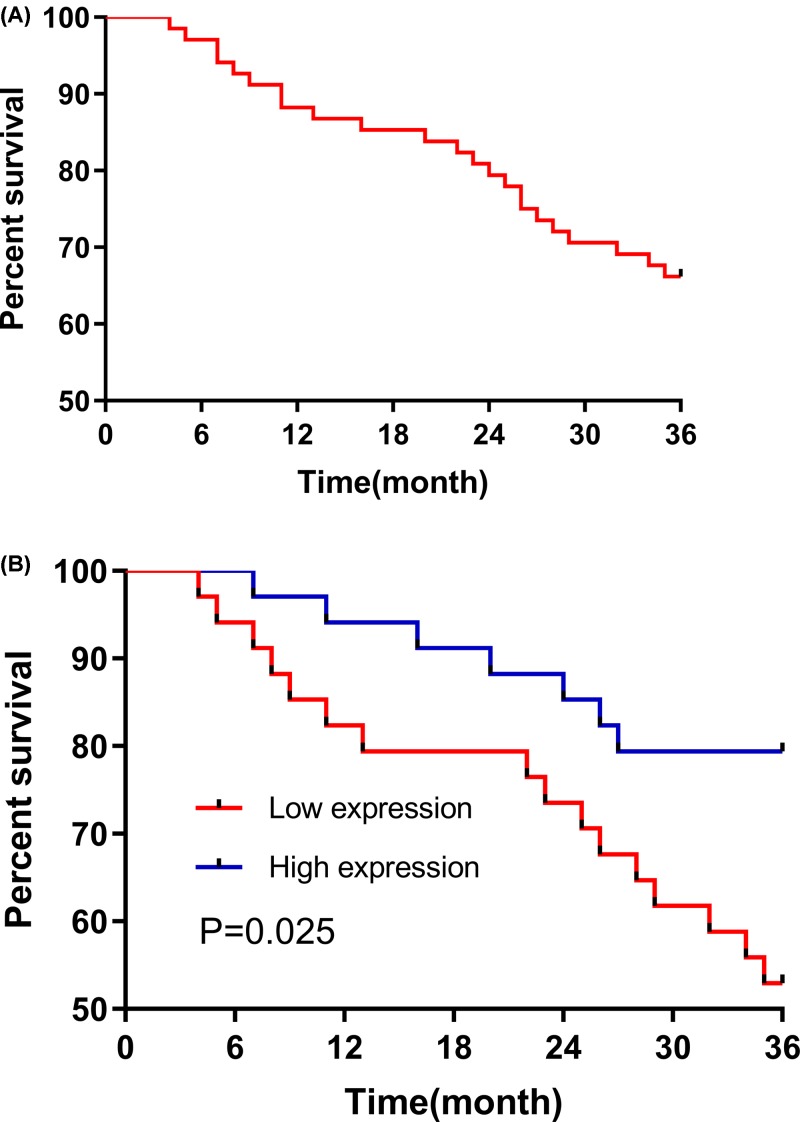
Three-year survival (**A**) It is the 3-year survival curve for all the CC patients (*n*=68). (**B**) The 3-year survival rate in the CASP12 low expression group (*n*=34) was significantly worse than in the CASP12 high expression group (*n*=34; *P*=0.028).

All CC patients (*n*=68) were followed for 3 years, and the 3-year survival rate was 73.5%. Then the patients were divided into two groups: a high CASP12 expression group (above the median CASP12 expression, *n*=34) and a low CASP12 expression group (below the median CASP12 expression, *n*=34). The results of the Kaplan–Meier analysis and log-rank test indicated that the 3-year survival in the CASP12 low expression group was significantly worse than in the CASP12 high expression group (*P*=0.028). More details are shown in Figure 5.

## Discussion

The incidence of CC, a common malignant tumor of the female reproductive system, is second only to breast cancer among female malignant tumors [14]. The disease is becoming more and more common in young females, which poses a severe threat to females’ health and their quality of life [15,16]. Early diagnosis and treatment are conducive to treatment. For example, previous studies have shown that patients with CC have a high postoperative survival rate if diagnosed early, but they are usually diagnosed in the middle and advanced stages, thus missing the best treatment timeframe [17,18]. Therefore, early diagnosis and screening are extremely important.

More and more gene sequencing programs have been launched to improve next-generation sequencing technology. The TCGA database, which is the most widely used, detects the key genes of 33 cancers comprehensively and draws multidimensional maps [19,20]. In this study, 306 CC samples and 3 matched paracancerous samples from the TCGA database were used to screen out potential differential protein-coding genes and observe their prognostic and diagnostic values. A total of 590 differentially expressed genes were found, 180 were highly expressed, and 410 were lowly expressed. Differential gene expression matrix files were established according to the screened differentially expressed genes and the clinical data of the samples, and then subjected to univariate and multivariate Cox regression analyses. The univariate analysis showed 36 genes with differences. Protein genes were screened, but not pseudogenes and long non-coding RNA were eliminated. According to the multivariate analysis, PDE2A and CASP12 were independent prognostic factors for CC. Additionally, there was no difference in the survival between the PDE2A groups with high and low expression, whereas the survival in the CASP12 low expression group was significantly worse than in the CASP12 high expression group. In summary, CASP12 was dramatically dysregulated in CC tissues through the TCGA database, and it was further proven to serve as a potential prognostic factor using the univariate (*P*=0.002) and multivariate Cox regression analysis (*P*=0.004). The data of the survival curve revealed a significant difference between the CASP12 groups with high and low expression (*P*=0.033). Therefore, we chose CASP12 for follow-up experiments to verify its potential prognosis value in CC.

CASP12, a cysteine protease [21], is widely expressed in 14 tissues such as the ovaries and endometrium and is highly correlated with members of the ICE subfamily [IL-1 invertase: IL-1β-converting enzyme (ICE)] that treats inflammatory cytokines [22,23]. In a study by Marshall et al. [24], the expression of CASP12 was significantly down-regulated during an infection, and there are protective factors between its polymorphism and rheumatoid arthritis. Therefore, CASP12 role in inflammation is widely known [25]; however, there are currently few studies on CASP12 in cancers. According to Long et al. [26], the high expression of CASP12 promotes apoptosis of intestinal epithelial cells, indicating that rapid proliferation of tumor cells could be related to the inhibition of CASP12 activity. In a study by Chu et al. [27], inhibition of CASP12 expression causes an increase in the invasiveness of nasopharyngeal carcinoma cells, and in a study by Cheng et al. [28], overexpression of CASP12 inhibits the proliferation of hepatoma HEP-J5 cells. Currently, there is no study on the correlation of CASP12 with CC. Therefore, the expression of serum CASP12 mRNA in patients with CC and healthy people were assessed in the present study, which was easy to operate. Furthermore, it provides potential diagnostic and prognosis indicator for CC. In the present study, the relative expression of CASP12 mRNA in the control group (healthy people) was significantly higher than in the experimental group (patients with CC), and the AUC of CASP12 was 0.865, revealing that CASP12, which has a high diagnostic value for CC and high sensitivity, has potential to become a diagnostic indicator for CC.

In the present study, there were statistically significant differences between the groups of CASP12 mRNA with high and low expression in terms of differentiation, lymph node metastasis, tumor size, FIGO staging and clinical outcomes. The 3-year survival rate in the CASP12 mRNA high expression group was significantly higher than in the CASP12 mRNA low expression group. These findings suggest that CASP12 can be used as a prognostic indicator in patients with CC. In the present study, the expression and clinical value of CASP12 in CC were verified through the TCGA database and clinical experiments. However, the correlation of CASP12 with the development and progression of CC remains unclear, which is the direction of future research. It is expected that the mechanism of action of CASP12 on CC could be explored through basic experiments, and to confirm the findings of this experiment. In summary, data mining and clinical experiments revealed and verified the differential expression of CASP12 in CC, and the expression level of CASP12 can be used as a diagnostic and prognostic biomarker in patients with CC.

## Abbreviations

AUC: area under curve
CASP12: caspase 12
CC: cervical cancer
FIGO: The International Federation of Gynecology and Obstetrics
HPV: Human papillomavirus
PDE2A: phosphodiesterase 2A
qRT-PCR: Quantitative real time polymerase chain reaction
ROC: Receiver Operating Characteristic
TCGA: The Cancer Genome Atlas
95CI%: 95%confidence interval

## References

[1] Dai S., Lu Y., Long Y. et al. (2016) Prognostic value of microRNAs in cervical carcinoma: a systematic review and meta-analysis. Oncotarget 7, 35369 10.18632/oncotarget.9294 27177085 PMC5085235

[2] Bray F., Ferlay J., Soerjomataram I. et al. (2018) Global cancer statistics 2018: GLOBOCAN estimates of incidence and mortality worldwide for 36 cancers in 185 countries. CA Cancer J. Clin. 68, 394–424 30207593 10.3322/caac.21492

[3] Chen W., Zheng R., Baade P.D. et al. (2016) Cancer statistics in China, 2015. CA Cancer J. Clin. 66, 115–132 26808342 10.3322/caac.21338

[4] Chen Y. (2016) Screening and risk assessment of early-stage cervical cancer in the health examination population. Ethics 10, 246–250

[5] Karuri A.R., Kashyap V.K., Yallapu M.M. et al. (2017) Disparity in rates of HPV infection and cervical cancer in underserved US populations. Front. Biosci. 9, 254 10.2741/s486 28410118 PMC5935458

[6] Sert B.M., Boggess J.F., Ahmad S. et al. (2016) Robot-assisted versus open radical hysterectomy: a multi-institutional experience for early-stage cervical cancer. Eur. J. Surg. Oncol. 42, 513–522 10.1016/j.ejso.2015.12.014 26843445

[7] Shazly S.A.M., Murad M.H., Dowdy S.C. et al. (2015) Robotic radical hysterectomy in early-stage cervical cancer: a systematic review and meta-analysis. Gynecol. Oncol. 138, 457–471 10.1016/j.ygyno.2015.06.009 26056752

[8] Heather J.M. and Chain B. (2016) The sequence of sequencers: the history of sequencing DNA. Genomics 107, 1–8 10.1016/j.ygeno.2015.11.003 26554401 PMC4727787

[9] Bleidorn C. (2016) Third-generation sequencing: technology and its potential impact on evolutionary biodiversity research. Syst. Biodivers. 14, 1–8 10.1080/14772000.2015.1099575

[10] Sanchez-Vega F. et al. (2018) Oncogenic signaling pathways in The Cancer Genome Atlas. Cell 173, 321–337 10.1016/j.cell.2018.03.035 29625050 PMC6070353

[11] Tomczak K., Czerwińska P. and Wiznerowicz M. (2015) The Cancer Genome Atlas (TCGA): an immeasurable source of knowledge. Contemp. Oncol. 19, A68 25691825 10.5114/wo.2014.47136 PMC4322527

[12] Javadi S., Ganeshan D.M., Qayyum A. et al. (2016) Ovarian cancer, the revised FIGO staging system, and the role of imaging. Am. J. Roentgenol. 206, 1351–1360 10.2214/AJR.15.15199 27042752

[13] Wang Y., Zhang J., Li L. et al. (2016) Identification of molecular targets for predicting colon adenocarcinoma. Med. Sci. Monit. 22, 460 26868022 10.12659/MSM.895881 PMC4754092

[14] Pareja R., Echeverri L. and Rendon G. (2018) Radical parametrectomy after ‘cut-through hysterectomy in low-risk early-stage cervical cancer: time to consider this procedure obsolete. Gynecol. Oncol. 149, 520–524 10.1016/j.ygyno.2018.02.015 29482838

[15] Cohen P.A., Jhingran A., Oaknin A. et al. (2019) Cervical cancer. Lancet North Am. Ed. 393, 169–182 10.1016/S0140-6736(18)32470-X 30638582

[16] Pesola F., Ferlay J. and Sasieni P. (2017) Cancer incidence in English children, adolescents and young people: past trends and projections to 2030. Br. J. Cancer 117, 1865 10.1038/bjc.2017.341 29096400 PMC5729467

[17] Yahata H., Sonoda K., Yasunaga M. et al. (2018) Surgical treatment and outcome of early invasive adenocarcinoma of the uterine cervix (FIGO stage IA1). Asia Pac. J. Clin. Oncol. 14, e50–e53 10.1111/ajco.12691 28429457

[18] Chen L., Keating N.L., del Carmen M.G. et al. (2018) Comparative effectiveness of minimally-invasive staging surgery in women with early-stage cervical cancer. Gynecol. Oncol. 149, 245–246 10.1016/j.ygyno.2018.04.553

[19] Akbani R., Levine D.A. et al. 2016 Abstract 133: Integrated molecular characterization of uterine carcinosarcoma in The Cancer Genome Atlas (TCGA) project, Cancer Research 76, 133–133 10.1158/1538-7445.AM2016-133

[20] Anaya J. (2016) OncoLnc: linking TCGA survival data to mRNAs, miRNAs, and lncRNAs. PeerJ. 2, e67 10.7717/peerj-cs.67

[21] Man S.M. and Kanneganti T.D. (2016) Converging roles of caspases in inflammasome activation, cell death and innate immunity. Nat. Rev. Immunol. 16, 7 10.1038/nri.2015.7 26655628 PMC4915362

[22] Fagerberg L., Hallström B.M., Oksvold P. et al. (2014) Analysis of the human tissue-specific expression by genome-wide integration of transcriptomics and antibody-based proteomics. Mol. Cell. Proteomics 13, 397–406 24309898 10.1074/mcp.M113.035600 PMC3916642

[23] Hermel E. and Klapstein K.D. (2011) A possible mechanism for the maintenance of the deleterious allele of human CASPASE-12. Med. Hypotheses 77, 803–806 10.1016/j.mehy.2011.07.041 21872999

[24] Marshall L., Obaidullah M., Fuchs T. et al. (2014) CASPASE-12 and rheumatoid arthritis in African-Americans. Immunogenetics 66, 281–285 10.1007/s00251-014-0762-9 24515649 PMC4139147

[25] Shalini S., Dorstyn L., Dawar S. and Kumar S. (2015) Old, new and emerging functions of caspases. Cell Death Differ. 22, 526–539 10.1038/cdd.2014.216 25526085 PMC4356345

[26] Long M., Chen X., Wang N. et al. (2018) Proanthocyanidins protect epithelial cells from zearalenone-induced apoptosis via inhibition of endoplasmic reticulum stress-induced apoptosis pathways in mouse small intestines. Molecules 23, 1508 10.3390/molecules23071508 PMC6099583 29933637

[27] Chu W.K., Hsu C.C., Huang S.F. et al. (2017) Caspase 12 degrades IκBα protein and enhances MMP-9 expression in human nasopharyngeal carcinoma cell invasion. Oncotarget 8, 33515 10.18632/oncotarget.16535 28380444 PMC5464886

[28] Cheng C.Y. and Su C.C. (2010) Tanshinone IIA inhibits Hep-J5 cells by increasing calreticulin, caspase 12 and GADD153 protein expression. Int. J. Mol. Med. 26, 379–385 20664954

